# Experiences of People With Long COVID Accessing Rehabilitation Services: A Qualitative Study

**DOI:** 10.1155/oti/4676712

**Published:** 2026-05-17

**Authors:** Daria St-Jean, Stephanie Haynes, Venezia Ficara, Anna Streib, Fatimata Ouédraogo, Anne-Marie Spiridigliozzi, Rosa Minichiello, Debbie Ehrmann Feldman, Barbara Mazer

**Affiliations:** ^1^ School of Physical and Occupational Therapy, Faculty of Medicine and Health Sciences, McGill University, Montreal, Quebec, Canada, mcgill.ca; ^2^ Centre for Interdisciplinary Research in Rehabilitation (CRIR), Montreal, Quebec, Canada, crir.ca; ^3^ Saint-Anne′s Hospital (CIUSSS de l′Ouest-de-l′Ile-de-Montreal), Sainte-Anne-de-Bellevue, Quebec, Canada; ^4^ Ergo-Medic Rehabilitation Center, Verdun, Quebec, Canada; ^5^ Jewish Rehabilitation Hospital, Centre intégré de santé et de services sociaux de Laval, Laval, Quebec, Canada, hjr-jrh.qc.ca; ^6^ CBI Health-Concordia Physio Sport, Lasalle, Quebec, Canada; ^7^ School of Rehabilitation, Faculty of Medicine, Université de Montréal, Montreal, Quebec, Canada, umontreal.ca; ^8^ School of Public Health, Université de Montréal, Montreal, Quebec, Canada, umontreal.ca; ^9^ Public Health Research Centre (CReSP), Montreal, Quebec, Canada

**Keywords:** access, healthcare, long COVID, rehabilitation services, resources

## Abstract

**Purpose:**

Long COVID is associated with a range of physical, cognitive, and/or psychological symptoms that significantly affect daily functioning. These individuals require rehabilitation services to address their limitations. This study explored the experiences of people with long COVID regarding access to and receipt of rehabilitation services.

**Methods:**

This qualitative study recruited 12 individuals with long COVID from a population‐based survey among the population of Laval (Quebec). Semistructured telephone interviews were conducted, recorded, transcribed verbatim, and analyzed using inductive thematic content analysis. Relevant elements were extracted, coded, and organized into themes/subthemes.

**Results:**

Twelve participants (seven females; five males) participated in the study. We identified three main themes, each with subthemes: (1) impact of long COVID on personal and professional life (e.g., professional life, leisure activities, and social life); (2) rehabilitation services (access barriers, perceptions of services received); and (3) psychosocial support (lack or presence of support). Access to rehabilitation services was hampered by several barriers: difficulties obtaining referrals, financial constraints, lack of awareness among health professionals, and service shortages. Participants who accessed rehabilitation services reported satisfaction with care they received and appreciated the multidisciplinary approach.

**Conclusions:**

Improving access to rehabilitation services for people with long COVID is essential to support their recovery, as timely and coordinated rehabilitation can facilitate reintegration into daily activities and reduce functional limitations. Strategies to enhance access include increasing health professional awareness, reducing financial and logistical barriers, and expanding service availability.

## 1. Introduction

Since its emergence in December 2019, COVID‐19, caused by the SARS‐CoV‐2 virus, has not only resulted in millions of deaths worldwide but has also left a growing population with persistent symptoms known as “long COVID” or “post‐COVID‐19 condition” [1]. According to the World Health Organization (WHO), long COVID occurs in individuals with a history of probable or confirmed SARS‐CoV‐2 infection, usually 3 months after the initial onset of COVID‐19, with symptoms lasting at least 2 months and not explained by another diagnosis [1]. A complementary definition is proposed by the National Academies of Sciences, Engineering, and Medicine (NASEM), according to which long COVID is an infection‐associated chronic condition that occurs following SARS‐CoV‐2 infection and persists for at least 3 months, presenting as a continuous, relapsing and remitting, or progressive disease state affecting one or more organ systems [2]. Worldwide, it is estimated that between 325 and 606 million people may be living with long COVID [3]. In Canada, approximately 3.5 million adults have experienced long COVID, with about 2.1 million individuals continuing to report persistent symptoms as of June 2023, according to Statistics Canada [4].

Symptoms of long COVID are complex and varied, with over 200 different manifestations that can vary from person to person [5]. The most common symptoms include fatigue, breathlessness, and cognitive dysfunction [1]. Recent studies conducted in Quebec with a sample of 121 participants with long COVID found similar symptoms [6, 7], as well as significant functional limitations [8]. The co‐occurrence of physical and psychological symptoms of long COVID has a substantial impact on the personal and professional lives of individuals [8], affecting their well‐being and quality of life [9–11]. For example, performing basic activities of daily living (ADL) (e.g., personal care) and instrumental activities of daily living (IADL) (e.g., groceries, food preparation, housework) which require increased physical and cognitive effort, often exacerbated patients′ symptoms, leading them to avoid participating in these activities, leading them to pace themselves or adjust their engagement in activities to prevent overexertion [12, 13]. This exacerbation reflects post‐exertional malaise (PEM), a hallmark feature of long COVID, whereby symptoms worsen after physical or cognitive exertion [14]. Statistics Canada estimates that in June 2023, more than one in five Canadian adults with these long COVID symptoms had to miss work or school, representing approximately 600,000 people and a total of 14.5 million days of absence [4].

Given the impact of physical and/or cognitive symptoms on patients′ functional capacity, it is clear that rehabilitation is a key element in improving the physical and emotional recovery of people with long COVID [11, 15–17]. This is consistent with WHO guidelines, which identify rehabilitation as an essential component of care for post‐COVID‐19 condition and recommends early, multidisciplinary, and individualized rehabilitation approaches [18].Rehabilitation can support the management of persistent symptoms such as breathlessness, fatigue, and PEM [16, 17, 19, 20]. It may also contribute to improvements in cardiac deconditioning, orthostatic intolerance (postural orthostatic tachycardia syndrome), psychological distress, sleep disturbances, and musculoskeletal pain [16, 17, 21]. For example, a few studies have reported improvements in physical symptoms, such as reduced breathlessness and fatigue, as well as enhanced emotional well‐being, characterized by increased self‐confidence and reduced anxiety [17, 22]. In addition, there is an expressed need for social support among people with long COVID, including empathy and recognition of the severity of their symptoms from healthcare professionals and family members [10].

Despite the evidence supporting the importance of rehabilitation in the recovery from long COVID symptoms, various barriers have limited access to appropriate rehabilitation services [23–25]. Rehabilitation services refer to any type of rehabilitation provided by rehabilitation professionals (e.g., physiotherapy and occupational therapy), whether delivered face‐to‐face in a clinical setting or remotely in the form of telerehabilitation. The barriers to getting rehabilitation services included high demand, insufficient service capacity relative to demand, long waiting times, and cancelations or rescheduling of appointments [23, 24]. Lack of knowledge about long COVID among healthcare professionals is also a barrier [10, 24, 26]. Conversely, a multidisciplinary and patient‐centered approach can serve as a facilitator by enabling coordinated and individualized care that addresses the multidimensional nature of long COVID, supports both physical and psychological recovery, and enhances patients′ engagement in rehabilitation services [18, 25].

Use of rehabilitation services by people with long COVID has been documented in several quantitative studies, providing valuable insights into general patterns of multiple symptoms and functional limitations, characteristics of treatment, and PEM [5–8, 21]. However, qualitative investigations could provide an in‐depth exploration of the experiences, perceptions, and expectations of people living with long COVID in relation to accessing and engaging in rehabilitation services. Although rehabilitation could be a key component of recovery, few qualitative studies have specifically addressed patient experience regarding access to rehabilitation services [27]. Therefore, the aim of this study was to explore the experiences of people living with long COVID regarding access to and receipt of rehabilitation services to inform the improvement of rehabilitation care and service delivery.

## 2. Methods

### 2.1. Study Design

We designed a descriptive qualitative study using semistructured telephone interviews to explore participants′ experiences with accessing and receiving rehabilitation services [28, 29]. We used thematic content analysis, with an inductive approach, allowing themes to emerge directly from the data without imposing a pre‐existing framework [30, 31]. The consolidated criteria for reporting qualitative research (COREQ) checklist was used as a reporting guideline [32].

### 2.2. Participants and Recruitment

The participants in this study were individuals with long COVID who were recruited from a previous survey on the use of rehabilitation services by people with long COVID among the population of Laval (Quebec, Canada) [7, 8]. This survey conducted between June and October 2022 was not part of this current study, and participants were not known to the researchers prior to recruitment to this study. The survey participants (*n* = 1549) who consented to be contacted for participation in future studies were eligible for inclusion in this qualitative study. The inclusion criteria for this study were as follows: (1) having had long COVID symptoms (other than loss of smell/taste as this symptom alone typically does not require rehabilitation services and was therefore outside the scope of the study), either currently or in the past, and (2) having sought out rehabilitation services. All eligible participants were classified into six categories based on sex (male; female) and age group (< 45 years, 45–65 years, > 65 years) to guide participant selection and ensure representation from various age and sex groups. Participants from each group were randomly selected, contacted and screened for eligibility until two participants from each group were recruited. If the list for a particular category was exhausted, an additional (third) respondent was recruited from another category to reflect the male/female ratio observed in people with long COVID. A research assistant contacted potential participants by telephone to explain the purpose of the research and to invite them to participate in an interview. Participants who expressed an interest in participating were then read a consent form, and those who gave verbal consent were given an appointment for a telephone interview in the following week, according to their availability. According to a systematic review, data saturation can be achieved with a sample size of 9 to 17 individual interviews [33]. After thorough discussion, the research team determined that data saturation was reached following the completion of 12 interviews, as no new codes, themes, or relevant insights emerged after the tenth interview.

### 2.3. Data Collection

Sociodemographic data and clinical information (e.g., age, sex, primary symptoms, and rehabilitation services received) were collected based on the results from the survey. Telephone interviews were conducted in either English or French depending on the participant′s preference, by either D.S‐J. or S.H., between June and August 2023. It should be noted that these interviews capture participants′ experiences of long COVID rehabilitation at a specific point in the pandemic (June–August 2023), reflecting the healthcare context and service availability during that period. The interview guide was developed by the research team based on the study objectives and existing literature on long COVID and access to rehabilitation services and was piloted on one person living with long COVID. It consisted of a wide range of open‐ended questions designed to explore participants′ experiences, with two to seven probes accompanying each question to facilitate in‐depth discussion. The guide was reviewed by team members with expertise in qualitative research and rehabilitation, and minor refinements were made following the first interviews to improve clarity and flow. The final interview guide is provided as supporting information (available here). For example, an initial question focused on persistent symptoms of long COVID and their functional impact (“Do you still experience symptoms of long COVID and how have they evolved over time?”; “Can you describe these symptoms and their main functional consequences in your daily life?”). Another question explored access to rehabilitation services, whether provided face‐to‐face in a clinical setting and/or remotely in the form of telerehabilitation (“Did you have access to rehabilitation services for these symptoms?”; “Can you describe any challenges or barriers you faced in the process of accessing these services?”; “Can you describe any treatment provided?”). One question addressed patient satisfaction with the rehabilitation services received (“On a scale of 0 to 10, how satisfied are you with the services you received?”). This quantitative item was included to provide contextual descriptive information and to support discussion during the qualitative interview. Another question explored psychosocial support (“Have you received any support services due to your long‐term COVID symptoms from friends, family or others?”). Each interview, lasting between 30–60 min, was recorded using digital audio recorders and initially transcribed with the dictation function in Microsoft Word. The transcripts were then manually reviewed by the two interviewers (D.S‐J. and S.H.) to correct any errors and to accurately identify the speakers throughout the transcripts. Transcription was conducted in “full verbatim” to preserve the participants′ responses and maintain objectivity. Each transcript was audited by the interviewer who conducted the specific interview.

The research team consisted of two researchers and five graduate students in rehabilitation. The two researchers each had a PhD in epidemiology, and had experience in qualitative research, expertise in long COVID research, and a background in occupational therapy (BM) or physiotherapy (DEF). All members of the research team were bilingual (English and French).

### 2.4. Data Analysis

Sociodemographic data and clinical information were analyzed using IBM SPSS Statistics Version 29.0.0.0 with descriptive statistics (means, standard deviations). Qualitative data analysis was performed using thematic content analysis, with an inductive approach [30, 31]. Content analysis is a systematic method of analyzing communication, whether written or verbal, and is particularly suited to exploring the experiences, reflections, and attitudes of an individual or a group of individuals [34, 35]. This method of analysis is therefore best suited to providing an in‐depth understanding of the experiences of people with long COVID regarding their access and receipt of rehabilitation services. An inductive content analysis approach was chosen as the aim of this study was to provide a descriptive and practice‐oriented understanding of participants′ experiences, making this method particularly appropriate for informing service development and clinical practice. Several steps were taken in the analysis. First, every transcription was read thoroughly many times by the members of the research team in order to obtain a sense of the whole. Second, the segments of the transcripts related to access to rehabilitation services were extracted and compiled into a single text (in a table), thus forming the unit of analysis. Third, the unit of analysis was divided into units of meaning, each consisting of several words, phrases, sentences or paragraphs containing aspects that are related by their content and context. Fourth, the units of meaning were condensed and independently coded in Excel by two members (D.S‐J. and S.H.), providing a structured yet flexible approach to data organization and analysis. The other team members then reviewed the codes, and several meetings were held to compare coding across interviews, resolve discrepancies, and reach consensus, thereby supporting investigator triangulation. The most frequently used codes included impact of long COVID, accessibility, and support. Finally, through a process of reflection and discussion, the authors re‐examined these codes, compared for differences and similarities, and regrouped to create themes and subthemes that aligned with the common patterns identified in the transcripts. This led to the identification of three main themes and six subthemes. Through this rigorous and iterative process of analysis, we ensured that themes emerged inductively from the data, rather than being predetermined by the interview questions.

### 2.5. Scientific Rigor

The methodology used in this study ensured scientific rigor, such as credibility, transferability, dependability, and confirmability, as identified by Lincoln and Guba [36]. The credibility and confirmability of the results were ensured through triangulation during data collection and analysis, as recommended by Lincoln and Guba [36] and Pourtois [37]. Indeed, this present study employed multiple triangulation strategies, including verification of initial transcriptions by two team members, followed by rechecking and validation by other members of the research team. Data coding was also carried out by the first two authors and then validated by the other team members. This approach minimized bias and strengthened the robustness of the findings. Reflexivity was also incorporated through summary sheets in which interviewers noted their personal impressions, which were later discussed within the team. This allowed for critical self‐assessment by the researchers of potential subjective influences on the interpretation of the results, further enhancing the confirmability of the findings. In addition, through the use of the COREQ checklist, our study provided a detailed description of the methods used, the study context, and the characteristics of the research team members. Participant quotations were used to illustrate the findings and enhance credibility by ensuring that interpretations were grounded in participants′ own perspectives and experiences. These methodological details enhance the scientific rigor of our research and provide readers with a clear understanding of the approach that led to our findings [38, 39].

### 2.6. Ethical Considerations

This study received ethics approval from the comité scientifique et d’éthique de la recherche de CISSS Laval (Ethics Approval Number 2022‐734). All participants reviewed the informed consent form and gave verbal consent prior to participating in the study. Participant anonymity was maintained by assigning an identification number to each participant. Interview responses were aggregated to ensure that no individual participant could be identified.

## 3. Results

Twelve persons participated in this study: seven females and five males. Although we intended to balance the sex distribution, when we were unable to find a participant in a specific category, we approached potential participants from other categories. Mean age of participants was 46.08 ± 15.25 years (range 24–66 years). Only one person had been hospitalized due to COVID‐19 infection. Table 1 describes the demographic and clinical information of the participants.

**Table 1:** Clinical and demographic characteristics of participants.

ID	Sex	Age (years)	Leading symptoms	Hospitalized for COVID‐19	Received medical services	Received rehabilitation services	Types of rehabilitation services received	Desired additional services	Types of additional services desired
1	F	27	Fatigue SOB Palpitations Dizziness	No	Yes	No	NA	Yes	Psych
2	F	47	Fatigue Brain fog Decreased Sensation Insomnia Depression	Yes	Yes	Yes	PT OT	Yes	Psych
3	M	58	Dizziness Headache Cognitive difficulties Fatigue SOB	No	Yes	Yes	PT OT Acup Kines Osteo	Yes	Psych
4	F	34	SOB Dizziness Visual disturbances Muscle weakness	No	Yes	Yes	PT OT	No	NA
5	M	28	Lung pain Fatigue SOB	No	Yes	No	NA	No	NA
6	M	69	Cognitive difficulties Fatigue SOB	No	Yes	No	NA	No	NA
7	F	24	Migraine Cognitive difficulties SOB Fatigue	No	Yes	No	NA	Yes	OT
8	F	53	Fatigue Brain fog Cognitive difficulties Muscle soreness	No	Yes	Yes	PT OT	Yes	SW
9	M	41	Insomnia Depression Cognitive difficulties Muscle soreness	No	Yes	Yes	PT	Yes	Psych
10	F	52	Fatigue SOB Dizziness Cognitive difficulties Muscle soreness	No	Yes	Yes	PT OT Acup	Yes	Chiro SW
11	M	54	Headache Persistent cough	No	No	No	NA	No	NA
12	F	66	SOB Fatigue Headache	No	Yes	No	NA	No	NA

Abbreviations: Acup, acupuncture; Chiro, chiropractor; Kines, kinesiology; NA, not applicable; Osteo, osteopathy; OT, occupational therapy; Psych, psychotherapy; PT, physiotherapy; SOB, shortness of breath; SW, social work.

### 3.1. Themes

Three main themes emerged from the interviews with participants: (1) impact of long COVID on personal and professional life; (2) rehabilitation services; and (3) psychosocial support. See Table 2 for quotes illustrating each theme. French quotes have been translated into English by the fifth author and verified by the two researchers of the research team.

**Table 2:** Thematic content analysis (*N* = 12).

Themes	Subthemes	*N*	%	Example quotations
Impact of long COVID on personal and professional life	Impact on professional life	12	100	ID8: “I forgot to mention that I am finishing my master′s degree at the University of Sherbrooke, and I was in the process of writing my thesis. So, I was writing, and I do not remember how much time I had left to present my work. And then, everything turned upside down last year, and I have not been able to write a single word in my thesis.” ID7: “I was working and in school at the same time, and I was overwhelmed. I could not remember anything. I was super slow, like, let us say I had an online class, I would have to repeat it… like I would have to re‐listen and re‐listen because it was like my brain just was not catching on. Again, the forgetfulness, like I would have a task to do at work, and I would just forget about it or, yeah, just… forget.”
	Impact on leisure activities	12	100	ID6: “It is certain that for a while, because of the fatigue and shortness of breath, I limited what I did. I was not engaging in physical activities beyond that; I did not take long walks. I just managed the daily routine but only did the minimum.” ID3: “It has impacted every part of my life because I cannot function like I used to. I mean, before, personally, I loved going biking and hiking. I started again this morning. I did a little 5 km. […] it is like a nice victory at the same time, but when I get back, yes, I am tired. And I cannot do a lot of things, but just one thing at a time, slowly.” ID1: “I used to be a very active athlete. I did a lot of running and all that. Then, it started happening more often… December… January… and I experienced a lot of fatigue in the following months. And it was when I started again in April, […] when I ran, I really felt unusual shortness of breath during exertion, palpitations. I had dizziness and extreme fatigue. I felt like I was experiencing tightness in my chest, actually.”
	Impact on social life	5	41	ID9: “It has definitely decreased, it has reduced my ability, and my energy, and my willingness to engage in social situations, my ability to sort of leave the home, riding in a car or vehicle is uncomfortable. So that has sort of reduce my radius. I am not going to visit family this year, despite them offered to literally fly me out, […], the last times I have done that, I have just ended up with more muscle aches and ended up in the emergency, and just yeah, it has not been a good thing.”
Rehabilitation services	**Barriers to accessing rehabilitation services:**			
	Referrals	5	41	ID10: “It seems that some long COVID patients have sleep apnea problems. So now I am going to do this at the beginning of July. I will have access to the tilt table at the [name of hospital]. I had to go through 2‐3 cardiologists to get this referral. […] I requested this referral from the first cardiologist last September and I got it on the 3rd of July”.
	Financial	5	41	ID4: “Since I fell at work, it went on CSST because I tripped over a small piece of carpet that nobody else trips over, but because I was so weak, I just crashed. So, I had physiotherapy 3 days a week for about a year […]. Alongside that, I also had occupational therapy for 2 days a week.” ID3: “The CNESST has helped me a lot by providing this service because I know it is expensive. Having access to these services makes me feel fortunate.”
	Service shortages	7	58	ID9: “Yeah, so there is definitely a, I would not say this other lack of service ‘cause obviously people are being served, but there is certainly a lack of volume of service.” ID3: “when I was at (Hospital name), they sent me a letter saying that I was to be followed by the …. Hospital in (town name). And then finally, I received a letter saying that it was no longer happening, and that it was canceled. It really complicated things for my follow‐up.”
	Lack of awareness and information	8	66	ID1: “It is certain that it was a difficult time because we do not really understand our symptoms; we do not know exactly… no one can confirm for us if it is really due to COVID.” ID2: “They need to train people. You know what I mean? That was it, like with the doctors” ID6: “The doctor ran tests to determine if it was potentially COVID because we knew very little about long COVID back in November 2021. It started with blood tests, then x‐rays of the lungs because I was easily short of breath. After that, I had a stress ECG for the breathing problems. Then, I also had a brain scan because I was having concentration issues. I also had an abdominal and pelvic MRI at the hospital to check if there was anything else wrong with my body. And they found nothing anywhere, and nothing justified my situation.”
	**Participants′ perceptions of the rehabilitation services received:**			
	Satisfaction with rehabilitation services	6	50	ID2: “Even though everything was online, I still have to say that it was effective and well done.” ID3:"Occupational therapy was really focused on my life, helping me understand how it could assist me in managing my efforts. […] So, occupational therapy is something that I would say I still need today.”
	Appreciation of the multidisciplinary approach	2	16	ID2: “There was a team that worked together, so the COVID clinic, even though everything was online, I must say it was effective and good. And the occupational therapists communicated with the physiotherapists, which was important. I do not know if it is still like that, but there was communication between the occupational therapists and the physiotherapists for the continuity of my services and what I needed.” ID3: “I think I have a good team around me. Yes, yes, because I look at how I was before, and I see how I am now. They listen, she listens, I feel the respect. That really touches me. And it is important to me because I do not know what my days are like, or what my tomorrows will be.”
	Segmented services	2	16	ID3: “With my psychotherapist, I am sending her documents via email from occupational therapy to inform her about what long COVID is, so she’s currently reading that.”
Psychosocial support	Positive aspect of receiving support	8	66	ID1: “But my family and my partner have been very supportive there.” ID4: “I literally wrote to my physiotherapists, both of them, last‐like 2 weeks ago to tell them thank you, because what I am doing today. I do not think would have been possible without them. The mental support that they give to their patients. I had two, no three very good people working with me, and the mental support was amazing.” ID7: “My mom like during my first weeks of COVID and then after that it was more like…just like reminders of like what I had to do and she would be like OK like what is your day like today? Just kind of telling me like OK like detail your days so I could try not to forget about anything, I got like an agenda, things like that.”
	Negative aspect of lack of support	4	33	ID8: “It is also the judgment around you from people who do not understand. Now with people coming out in the media, people are starting to understand. But before they called it long COVID, they thought I had COVID all the time, you know?” ID10: “Physiological, that is it. People think—some say it is psychological, but that is not it.” ID3: “Having someone who listens to me, someone who respects me, and listens to me. I say this because it is not always easy. The people you talk to when discussing long COVID, many of them will say yes, yes, yes. But they do not know. They do not understand what the topic is about.”

#### 3.1.1. Theme 1: Impact of Long COVID on Personal and Professional Life

Participants reported that symptoms of long COVID had a significant impact on their meaningful activities. Two subthemes emerged: (1) impact on professional life; and (2) impact on leisure activities and social life.

##### 3.1.1.1. Impact on Professional Life.

Relatively young and highly active, the impact of long COVID on participants′ working lives was profound and varied, affecting their ability to study and/or work effectively. All the participants reported a significant loss or decline in both cognitive and physical abilities, which prevented them from studying as they had previously. They also mentioned memory and concentration problems, to the extent that they had to repeat online courses several times in order to understand the information. This cognitive slowness and these memory problems were particularly challenging for those juggling studies and work, leaving them feeling overwhelmed and struggling to maintain performance in both areas. For example, ID7 said “I was working and in school at the same time, and I was overwhelmed. I could not remember anything. I was super slow, like, let us say I had an online class, I would have to repeat it […] like I would have to re‐listen and re‐listen because it was like my brain just was not catching on. Again, the forgetfulness, like I would have a task to do at work, and I would just forget about it or, yeah, just… forget*.”*

For participants, long COVID symptoms made it difficult, if not impossible, to perform their job duties. Participant ID2 mentioned his inability to work for more than three consecutive hours without experiencing severe effects. This lack of consistency directly affected his ability to manage his clients and maintain a stable work rhythm. He said *“*I am self‐employed, so I set my own hours, but I cannot manage. I work in finance and have a very large client base, but I am unable to manage everything. It is not fixed, it depends on what work I can do. […]. I can no longer work more than 3 h in a row without repercussions, sometimes even less.”

##### 3.1.1.2. Impact on Leisure Activities and Social Life.

Participants described their leisure activities as consisting exclusively of physical activities. According to them, the symptoms of long COVID had a profound impact on these activities, limiting their abilities and altering their routines. These activities included running, going to the gym, cycling, and taking long walks. The impact ranged from a complete cessation of physical activities to necessary adjustments to manage fatigue, pain, and other symptoms related to long COVID. For example, ID4 stated “The symptoms made many things difficult. I could not go to the gym anymore, even though I used to go four times a week. I really was not moving as much, and I gained a lot of weight.” On the other hand, some participants adjusted their physical activities to match their new physical capabilities, going at a much slower pace.

Moreover, the multiple symptoms of long COVID had a significant impact on participants′ social life. Fatigue and loss of energy reduced the participants′ ability and willingness to engage in social situations, leave their homes, and travel by car. This forced five participants to limit their social participation, even to the point of giving up visits to their families. For example, ID9 said “It has reduced my energy and willingness to engage in social situations, my ability to sort of leave the home. Riding in a vehicle is uncomfortable, […] and previous attempts have led to muscle aches and emergency visits, […] I am not going to visit family this year.”

The impact of these symptoms on participants′ meaningful activities led all of them to seek rehabilitation services. Participants expressed a strong desire to regain their previous physical, cognitive, and mental abilities, recover lost skills, reconnect with their “pre‐COVID self,” return to work or studies, and resume family responsibilities. For example, ID8 stated “It was about finding the [patient′s name] from before COVID. […] So, I wanted to regain some of my skills, my cognitive abilities, to at least be able to continue and return to work. My physical abilities, because I have a 14‐year‐old child and a 26‐year‐old daughter, I have my husband, we have a very active social life, but I was the driving force of the house and the family.”

#### 3.1.2. Theme 2: Rehabilitation Services

This theme is divided into two subthemes: (1) barriers to accessing rehabilitation services; and (2) participants′ perceptions of the rehabilitation services received.

##### 3.1.2.1. Barriers to Accessing Rehabilitation Services.

Participants reported several barriers to accessing rehabilitation services, including difficulties in obtaining referrals to the necessary services, financial constraints, a shortage of available services, and the lack of awareness and information among healthcare professionals.

• Referrals: Five participants reported difficulties in obtaining referrals to rehabilitation services, including initial refusals or long delays in obtaining the necessary referrals. This lack of responsiveness within the healthcare system caused considerable frustration and uncertainty for patients, ultimately impacting their recovery trajectory. They expressed feelings of abandonment, as they were left waiting for long periods before receiving necessary interventions. For example, ID10 said “I will have access to the tilt table at the [hospital]. I had to go through 2–3 cardiologists to get this referral. […]”
• Financial: Long COVID symptoms, which prevented participants from working fully or caused prolonged absences from work, had a significant financial impact. This contributed to increasing concern about existing economic difficulties, which were likely to worsen over time. For example, ID2 pointed out “Financially, the impact on my career… after a year and a half, it is really starting to become dangerous for my financial health.” Five participants reported financial barriers, such as the high cost of rehabilitation services not covered by insurance that limited their access to rehabilitation services. These barriers placed a heavy financial burden on individuals who were already dealing with the challenges of their health conditions. For example, ID9 explained the financial strain he experienced: “I am in there between the insured cost and the uninsured cost, I spent $4000 on physio last year”. Some participants felt fortunate that their insurance exempted them from these costs for certain services. However, for services not covered by insurance, participants were forced to space out sessions, even though they were essential to their well‐being, which limited the effectiveness of their treatment. ID3 stated “Of course, according to him (the osteopath), he would like to see me every 2 or 3 weeks, but it is because I cannot afford to pay for it […]. I have to space out [the sessions] because with insurance, at some point, I will not be able to afford it anymore.” Given that long COVID symptoms prevented people from working adequately, there was an impact on participants′ finances. The combination of reduced income and high out‐of‐pocket expenses for rehabilitation care made it difficult for participants to manage both their health needs and their financial situation. Using their limited income to pay for rehabilitation care was challenging for participants, even though they recognized the usefulness of these services. For example, ID9 highlighted “So that is that is very helpful, but it is unfortunately out of pocket and being unable to work and not having any revenue makes that harder to access.”
• Service shortages: Service shortages in rehabilitation led to long waiting lists, the need to travel long distances to access limited services, and the closure of some departments, further reducing the availability of care. In some cases, the closure of the rehabilitation services left participants without adequate follow‐up, leaving them on their own. Additionally, frequent changes in rehabilitation professionals also had a negative impact on the continuity of care. For example, ID8 explained “In terms of people, professionals, it was super good, 10/10. But it changed every 3 weeks. […]. So you would have someone for 2–3 weeks, and then she would be gone. That is why, as professionals, they were perfect, but as… how shall I put it? Not stability, but it lacked that continuity in follow‐up, which made things more difficult.”
• Lack of awareness and information: The lack of awareness and information among healthcare professionals regarding long COVID limited the access to rehabilitation services, according to eight participants. This lack of awareness led to a failure to recognize symptoms, resulting in repeated medical tests without a conclusive diagnosis and delaying access to appropriate rehabilitation care. For example, ID8 reported that her symptoms were initially interpreted as depression rather than manifestations of long COVID: “Last year, because, you know, at the beginning of COVID, not all doctors knew about long COVID and believed in it. So, the specialist said maybe I have depression and not COVID.” Therefore, all participants emphasized the importance of training health professionals in long COVID so that they can better understand and respond to rehabilitation needs. They stressed the need for resources such as occupational therapy and criticized the lack of adequate training for professionals, which led to under‐prescribing of these essential services. In addition, some participants were unaware of the existence of services such as occupational therapy that could help them manage their symptoms. When asked about her prior knowledge of occupational therapy, ID7 added “None at all. Like I had to Google it when I got home.”

##### 3.1.2.2. Participants′ Perceptions of the Rehabilitation Services Received.

###### 3.1.2.2.1. Satisfaction With Rehabilitation Services.

The rehabilitation services received varied between participants and included occupational therapy, physiotherapy, kinesiology, acupuncture, and osteopathy. Most of the rehabilitation services were provided through telerehabilitation, as mentioned by ID11 “I had the physiotherapy once a week. Then I also had the occupational therapy with the clinic. Both were online.” The six participants who had access to at least one of these services were satisfied with the care they received. On a scale of 0–10, participants′ satisfaction with each of the rehabilitation services they received ranged from 8 to 10. In response to the interviewer′s question, “On a scale of 0 to 10, how satisfied are you with this service (referring to occupational therapy)?” ID8 replied “10/10 for everything she has done.” Similarly, for the question, “And on a scale of 0–10, how would you rate your satisfaction with the physiotherapy service?” ID3 replied, *“*8.5–9.”

Six participants emphasized the important role of these rehabilitation services in managing long COVID symptoms. For example, ID2 emphasized the importance of occupational therapy in managing his symptoms. He stated, “Anyone with long COVID should have access to this type of specialist, which unfortunately is not the case. At least OT, especially OT […] I was not even aware of managing this before, so it gave me important tools. I still use them today.” Similarly, in response to the interviewer′s question, “And have you found that kinesiology has helped you with your long‐term COVID recovery to date?” ID3 explained how kinesiology had contributed to improving his condition. He replied, “Yes, yes, yes, yes, absolutely. Yes. Because at the beginning, […] I was not moving very much and then, […] for sure, now, yes, there has been an improvement. Like this morning, I went for a quiet 5 km bike ride.”

Two participants particularly appreciated the multidisciplinary approach among some rehabilitation professionals, which played a key role in the quality of their experience and was essential for ensuring continuity of care. This level of collaboration between professionals adequately addressed the specific needs of the participants.

###### 3.1.2.2.2. Segmented Services.

The segmentation of other services posed a limitation for participants with multiple symptoms, who would have preferred a multidisciplinary approach among services to facilitate the management of their impairments. Service segmentation referred to a healthcare organization in which different healthcare professionals (doctors, physiotherapists, occupational therapists, etc.) often work in isolation, in separate facilities, with no direct interaction or coordination between them. In contrast, a multidisciplinary approach involved active collaboration and continuous communication between different professionals to optimize patient care and rehabilitation. For example, ID9 stated “It is quite difficult to get into the public system for any kind of physiotherapy or rehabilitation, and even when you get there, it is highly compartmentalized into disciplines.”

#### 3.1.3. Theme 3: Psychosocial Support

To cope with the physical, cognitive, and psychological symptoms associated with long COVID, all participants emphasized the importance of psychological support provided by both family members and health professionals. Two subthemes were identified to describe this theme: (1) negative aspect of lack of support; and (2) positive aspect of receiving support.

##### 3.1.3.1. Negative Aspect of Lack of Support.

Four participants felt judged and misunderstood by those around them, who often gave them medical advice or diagnoses that invalidated their feelings, experiences, and condition. For example, ID8 said “Last year, because, you know, at the beginning of COVID, not all doctors knew about long COVID and believed in it. So, the specialist said maybe I have depression and not COVID”. Therefore, they expressed the need to be listened to, validated and encouraged. For example, ID7 said “ I knew that for my anxiety… my best option would be to like to talk to someone outside of my circle of friends, my family, someone who could just like validate the way I was feeling. That is what I needed at that time.”

##### 3.1.3.2. Positive Aspect of Receiving Support.

Eight participants appreciated the emotional support provided by rehabilitation professionals. Regardless of who provided the support, the encouragement, reassurance, and compassion received were deemed vital for their well‐being, and no amount of it was perceived as too much.

Moreover, the participants expressed that their families/partners were very important resources on which they could rely. They revealed that support from families and loved ones played a central role in managing the daily challenges of the disease and its physical and emotional impact. Participants received help with personal care, childcare, household responsibilities, meal preparation, and planning of professional activities. Some of them highlighted the invaluable support of their spouses, who, during the first few weeks of the illness, assumed additional responsibilities such as financial and household responsibilities that had previously been shared between both partners. For example, ID3 said “My spouse helped me through all of this. He was the key person, who supported me in this, at home, in my daily life at the beginning.”

Others benefited from family support, which proved essential in compensating for the severe physical and cognitive symptoms imposed by long COVID.

## 4. Discussion

The results of this qualitative study highlight the profound impact the multiple symptoms of long COVID had on the personal and professional lives of those affected. To manage these symptoms, many participants sought rehabilitation services. However, they encountered several major barriers limiting access to these services. Participants who accessed rehabilitation services were satisfied with the services they received, and particularly appreciated the multidisciplinary approach offered by some rehabilitation professionals. Furthermore, psychosocial support, both from family members and rehabilitation professionals, was deemed essential. The following discussion focuses on these key findings.

First, the wide range of symptoms including physical (fatigue, shortness of breath), and/or cognitive (difficulty concentrating, memory problems) and/or psychological (anxiety, depression) had a significant impact on the participants′ productive lives, leading to interruptions in training schedules and difficulties in attending courses for some participants who were juggling work and study, reduced working hours, and even needing to stop work altogether. This is understandable, as work or study capacity is a dynamic process in which the worker must be able to meet the demands of their tasks by using their physical, cognitive and mental abilities [40]. Therefore, due to the range of symptoms that affected the work or study capacity of people with long COVID, returning to work and/or study was a major challenge. This was highlighted by Hossain et al. in their systematic review of 15 qualitative studies [11], and aligns with the clinical practice guidance emphasizing that long COVID impacts patients′ multiple dimensions of participation and identity [41]. Our findings are also consistent with previous research which found that some participants took sick leave and doubted their ability to return to work, given their symptoms and the physical and mental demands of their jobs [13, 42]. Those who did return to work were unable to fully regain their work capacity, as they had to cope with both the challenges associated with their symptoms and the financial pressures resulting from a long absence [13, 42]. Similarly, previous studies have shown that attending classes was perceived as extremely difficult by participants and led to a decline in their academic performance [43, 44]. In this context, the implementation of accommodations, such as flexible remote working, adjustments to job responsibilities, and modifications to the work environment to improve access and engagement, is essential to enable individuals with long COVID to continue working or return to work [45]. Nonwork factors were also found to be essential, particularly the ability to obtain a long COVID diagnosis, which is key to accessing leave and necessary accommodations [45]. Increased support in the workplace, together with an empathetic attitude from colleagues, is important to facilitate return to work [11]. Similarly, the need for accommodation and understanding on the part of educational staff was also mentioned as essential for supporting participants who attend school [43, 44]. These findings suggest that rehabilitation professionals could support graded return‐to‐work or return‐to‐study planning and collaborate with employers and educational institutions to implement individualized accommodations aligned with patients′ functional capacities, in line with the clinical practice guidance, emphasizing that rehabilitation for long COVID should extend beyond symptom management to support engagement in meaningful social, occupational, and educational roles [41]. Given that the participants in this study were relatively young and highly active, such collaborative approaches may be particularly important to facilitate their reintegration into work and study and to maintain participation in valued roles.

In addition, participants reported that long COVID had an impact on their engagement in leisure activities, particularly physical activities. Reduced time spent in physical activity, changes, and stopping physical activity altogether were mentioned by participants. These changes led to weight gain in some, which can lead to other health problems, such as Type 2 diabetes, cardiovascular disease, and cancer, which are responsible for nearly three‐quarters of deaths worldwide, according to the WHO [46]. Large epidemiological studies have shown that physical inactivity (defined as weekly physical activity below WHO recommendations) is associated with an increased risk of hospitalization in people with COVID‐19. This highlights the importance of physical activity in the management of long COVID symptoms [47, 48]. These findings underline the role of rehabilitation in guiding gradual, patient‐centered re‐engagement in meaningful physical activities while balancing symptom management and energy conservation.

Second, the multiple impact of long COVID symptoms led participants to seek rehabilitation services to regain their pre‐COVID physical, cognitive, and psychological abilities. Previous studies have also reported accessing these services as a way to cope with impairments related to long COVID and the desire to regain pre‐COVID functional levels [12, 27]. However, we identified several barriers that limited participants′ access to rehabilitation services. Difficulty in getting referrals to rehabilitation professionals, despite repeated requests, was reported as a significant source of frustration. This is consistent with previous studies that reported similar frustrations among participants regarding GPs′ refusal to refer them to specialists [13, 42], leading some participants to resort to self‐management strategies, such as self‐prescribing vitamin supplements or changing their diet [13].

Our findings could be explained by a lack of awareness and information among healthcare professionals about the symptoms of long COVID and the rehabilitation services available. For example, due to the novelty of long COVID, many healthcare professionals may lack updated training or guidelines, hindering proper diagnosis and referral to rehabilitation services. Several previous studies have shown that knowledge gaps were a barrier to accessing specialist care, such as rehabilitation services [12, 13, 27, 49]. This may also depend on patients′ knowledge of these services [27]. For example, Hung et al. found that getting referrals for rehabilitation services depended on both practitioners′ and patients′ knowledge of these services [27]. In this study, some participants had not even considered asking for a referral to rehabilitation service because they did not think it could help them [27]. In addition to the lack of awareness among healthcare professionals, several systemic barriers in Canada including, but not limited to, limited capacity in specialized clinics to meet growing demand, complex administrative processes, and the concentration of services in urban centers, which makes access difficult for residents in rural or remote areas may also impede timely access to rehabilitation. Addressing both professional awareness and systemic factors can improve service delivery and patient outcomes.

Financial constraints were also a major barrier to accessing rehabilitation services that were not covered by insurance. Given the significant impact of the disease on participants′ work lives, some were already facing financial difficulties that made it challenging to afford the high cost of rehabilitation services. These financial difficulties could create a compounding cycle of strain, particularly for those who were already financially vulnerable. Limited capacity to afford private rehabilitation services could leave many without adequate support to manage their symptoms. Without access to rehabilitation, their ability to manage long COVID symptoms and regain functionality further deteriorates, perpetuating challenges in performing work duties and deepening their financial vulnerability. Brehon et al. also found that many participants explained their low income, resulting from their inability to work, prevented them from accessing services related to long COVID [12]. Another study conducted in Alberta (Canada) found that the use of personal savings to pay for private practitioners exacerbated the financial burden for people with long COVID [27].

Another barrier in our study was the shortage of rehabilitation services, which resulted in long waiting lists, the closure of some services leaving participants without support, and frequent changes in rehabilitation professionals negatively affecting the continuity of care. A recent systematic review of two qualitative and 28 quantitative studies highlighted the importance of continuity of care for participants with long COVID, who often had long medical histories [50]. Similar barriers have been reported by people with long COVID in previous studies, where long waiting times for appointments, the obligation to travel long distances, and the discomfort of commuting due to their symptoms were identified as major barriers to accessing rehabilitation [12, 23]. To address these barriers, a multifaceted approach is essential to improving access to rehabilitation services. For example, raising awareness among healthcare professionals and patients about long COVID symptoms and available rehabilitation services can ensure timely referral to public services or direct access to private options for those with the financial means to afford these services. Additionally, extending insurance coverage to all rehabilitation services could further reduce financial barriers and enhance accessibility. Telerehabilitation services can also improve access to rehabilitation services, reduce waiting times, and minimize the need for travel [51].

Third, participants in this study who did have access to rehabilitation services (e.g., physiotherapy, occupational therapy) expressed satisfaction with the services they received and appreciated the multidisciplinary approaches adopted by some rehabilitation professionals, which is consistent with previous studies [13, 27, 52]. For example, occupational therapy was perceived by people with long COVID as an essential support in managing their symptoms [13]. However, other participants complained about the fragmentation of services and emphasized the need for a multidisciplinary approach to help them manage their multiple symptoms. This was also highlighted by Kingston et al. in their qualitative study of 24 people with long COVID [10]. In our study, the majority of rehabilitation services were delivered remotely, which was much appreciated by the participants. Similarly, in the study of Brehon et al., participants expressed satisfaction with this approach as it helped to overcome several geographical and physical barriers, such as fatigue, pain and breathlessness, which would otherwise have prevented participants from traveling [12]. Aiyegbusi et al. also noted that participants valued virtual care options as they reduced face‐to‐face contact and the risk of reinfection [53]. Telerehabilitation can enhance access to rehabilitation services for certain patients but may also introduce barriers for individuals with limited digital access or technological literacy. It should therefore be considered as one component of a flexible rehabilitation approach rather than a universal solution [51].

Finally, the multiple symptoms of long COVID significantly reduced social participation, such as visiting family or friends, which could lead to social isolation. Similarly, Thomas et al. found that visiting family or participating in other social activities, such as attending church, was perceived by people with long COVID as exhausting and causing chest pain [13]. Thus, our results highlighted the importance of psychosocial support from both family members and healthcare professionals to manage emotional distress and to assist with performing ADL (personal care) and IADL (food preparation, housework, and groceries). This is consistent with previous studies, which have shown that many participants emphasized the importance of psychosocial support throughout their journey with long COVID, including support from spouses, friends, parents, and children, who helped with ADL and provided financial and emotional support [12, 13, 50]. Moreover, participants appreciated the psychological support they received from rehabilitation professionals. The need to be listened to attentively, without judgment, with empathy, to be believed, validated, and reassured was identified by all participants as essential to managing the multiple symptoms associated with long COVID, a finding that is echoed in previous studies [11, 12, 26, 50]. These findings highlight the importance of integrating psychosocial support into rehabilitation programs for people with long COVID to promote emotional well‐being and facilitate participation in daily and social activities.

Taken together, our findings resonate with the clinical practice guidance for long COVID rehabilitation, which emphasizes interdisciplinary, person‐centered care that supports participation in daily life, informed by an understanding of the complex and fluctuating nature of the condition [41]. Furthermore, monitoring the impact of long COVID on pre‐existing health conditions is important, as the interaction between chronic conditions and long COVID may influence symptom presentation, functional outcomes, and rehabilitation needs. This consideration is particularly relevant given that the WHO definition of long COVID excludes individuals with other pre‐existing conditions, highlighting a gap that warrants further exploration.

## 5. Strengths and Limitations of the Study

This study has methodological strengths that enhance the credibility of its findings. For example, it used multiple triangulation strategies [36, 37], including review of initial transcriptions by two team members, followed by review and validation by other members of the research team. Data coding was also carried out by at least two members of the research team. This approach minimized bias and strengthened the robustness of the findings. Reflexivity was also incorporated through summary sheets in which interviewers recorded their personal impressions, which were later discussed within the team. This allowed for critical self‐assessment by the researchers of potential subjective influences on the interpretation of the results, and further enhanced the confirmability of the findings. By using the COREQ checklist, our study provided a detailed description of the methods used, the study context, and the characteristics of the research team members. These methodological details enhanced the scientific rigor of our research and provided readers with a clear understanding of the approach that led to our findings. However, there were some limitations that should be noted. A limitation that could have affected interview responses was fatigue, and memory, word‐finding and concentration difficulties, as well as brain fog, which are characteristic of long COVID. In addition, as the interviews were performed over the phone, the transcripts did not include any nonverbal communication, which is an important element of narrative interviewing. This could have been rectified with in‐person or video call interviewing. Although the sample size was sufficient to reach data saturation, the heterogeneity of long COVID presentations means that this study may not capture the full range of symptoms, rehabilitation needs, and experiences of individuals living with long COVID. The resulting sample did not fully represent the female predominance reported in international studies. This should be considered when interpreting and transferring the findings to other contexts.

## 6. Conclusion

The findings of this study reveal that the various physical (fatigue, shortness of breath), cognitive (memory problems, difficulty concentrating), and psychological (anxiety, stress) symptoms associated with long COVID have significantly affected multiple aspects of the participants′ lives, including their professional and social lives, leisure activities, and economic situation. Faced with these new limitations, participants sought rehabilitation services to address these functional impairments. However, they encountered several barriers, such as difficulties obtaining referrals to specialized services, financial constraints to pay for services not covered by insurance, a lack of knowledge and information among healthcare professionals regarding long COVID, as well as service shortages leading to long waiting lists. Those who did receive rehabilitation services, including physiotherapy, occupational therapy, acupuncture, and kinesiology, expressed a high level of satisfaction, even when the services were provided through telerehabilitation. The multidisciplinary approach adopted by some professionals was particularly appreciated. Nevertheless, service fragmentation was perceived as a challenge, with participants preferring an integrated and centralized approach within a single healthcare facility. Finally, the social and emotional support provided by family members and healthcare professionals was highly valued. Participants expressed the need for specific training for health professionals on long COVID to facilitate quicker referral to appropriate rehabilitation services. Clinically, these findings highlight the need for healthcare systems to develop accessible, multidisciplinary, and integrated rehabilitation programs for individuals with long COVID, ensuring timely referrals, financial support, and professional training to optimize recovery and quality of life. They also indicate that rehabilitation professionals can support graded return‐to‐work or return‐to‐study planning and collaborate with employers and educational institutions to implement individualized accommodations aligned with patients′ functional capacities. Future studies could explore how sociodemographic factors such as age, sex, and clinical history impact patients′ experiences with accessing and receiving rehabilitation services, to better understand their influence on outcomes and clinical practices.

## Funding

This study was supported by Jewish Rehabilitation Hospital Foundation; Foundation Cité de la Santé.

## Conflicts of Interest

The authors declare no conflicts of interest.

## Supporting information


**Supporting Information** Additional supporting information can be found online in the Supporting Information section. The interview guide contains the open‐ended questions used to explore participants’ experiences along with the probing follow‐up questions.

## Data Availability

The data supporting the findings of this study are available from the corresponding author, [BM], upon reasonable request.
