# Supplementary material for: Experiences of People With Long COVID Accessing Rehabilitation Services: A Qualitative Study
Source: Occup Ther Int. 2026 May 17;2026:4676712. doi: 10.1155/oti/4676712 (PMC13181215; doi:10.1155/oti/4676712)
Supplement: Supplementary file 1 — Supporting Information Additional supporting information can be found online in the Supporting Information section. The interview guide contains the open‐ended questions used to explore participants’ experiences along with the probing follow‐up questions. [file OTI-2026-4676712-s001.docx]

1. **What are your long COVID symptoms, and how have they evolved over time?**

- Have you experienced any of the following common symptoms? Fatigue? Shortness of breath?
- Headaches or dizziness?
- Memory problems or difficulty finding words? Trouble concentrating?
- Digestive issues?
- Are you able to drive?
- What are the main functional impacts of these symptoms on your daily life?
- Could you specify if you had to take time off work? If you have returned to work, how has the transition been?

1. **Did you seek rehabilitation services such as physiotherapy, occupational therapy, speech therapy, or other similar services to manage your symptoms?**

- What was your knowledge of these rehabilitation services before receiving them for your long COVID?
- Could you describe any challenges or barriers you encountered when trying to access these services?
- What types of assessments or treatments were offered to you?
- What were your goals in using these rehabilitation services?

1. **On a scale of 0 to 10, how satisfied are you with the rehabilitation services you received?**

- Did these services help you recover from your long COVID?
- What do you think could be improved?
- Are there any services you would have liked to receive but were not available?

1. **Did you receive any support services for your long COVID symptoms, such as:**

- Meal delivery services, home assistance, or cleaning services?
- Support from friends or family?
